# Direct Oral Anticoagulants or Standard Anticoagulant Therapy in Fragile Patients with Venous Thromboembolism

**DOI:** 10.1055/s-0039-1683970

**Published:** 2019-03-20

**Authors:** Juan J. López-Núñez, Ricard Pérez-Andrés, Pierpaolo Di Micco, Sebastian Schellong, Covadonga Gómez-Cuervo, Joan Carles Sahuquillo, Maurizio Ciammaichella, Maria del Valle Morales, Marijan Bosevski, Manuel Monreal, and the RIETE Investigators

**Affiliations:** 1 Department of Internal Medicine, Hospital Universitari Germans Trias i Pujol, Universitat Autònoma de Barcelona, Badalona, Spain; 2 Department of Radiology, Hospital Universitari Germans Trias i Pujol, Universitat Autònoma de Barcelona, Badalona, Spain; 3 Department of Internal Medicine and Emergency Room, Ospedale Buon Consiglio Fatebenefratelli, Naples, Italy; 4 Department of Medical Clinic, Municipal Hospital of Dresden Friedrichstadt, Dresden, Germany; 5 Department of Internal Medicine, Hospital Universitario 12 de Octubre, Madrid, Spain; 6 Department of Internal Medicine, Hospital Municipal de Badalona, Barcelona, Spain; 7 Department of Emergency Internal Medicine, Ospedale St. John, Rome, Italy; 8 Department of Internal Medicine, Hospital del Tajo, Madrid, Spain; 9 University Cardiology Clinic, Faculty of Medicine, Skopje, Republic of Macedonia

**Keywords:** venous thromboembolism, fragile patients, direct oral anticoagulants

## Abstract

**Background** The efficacy and safety of the direct oral anticoagulants (DOACs) in fragile patients (age ≥ 75 years and/or creatinine clearance levels ≤ 50 mL/min and/or body weight ≤ 50kg) with venous thromboembolism (VTE) has not been evaluated.

**Methods** We used the RIETE database to compare the rates of the composite of VTE recurrences or major bleeding during anticoagulation in fragile patients with VTE, according to the use of DOACs or standard anticoagulant therapy.

**Results** From January 2013 to April 2018, 24,701 patients were recruited. Of these, 10,054 (41%) were fragile. Initially, 473 fragile patients (4.7%) received DOACs and 8,577 (85%) low-molecular-weight heparin (LMWH). For long-term therapy, 1,298 patients (13%) received DOACs and 5,038 (50%) vitamin K antagonists (VKAs). Overall, 95 patients developed VTE recurrences and 262 had major bleeding. Patients initially receiving DOACs had a lower rate of the composite outcome (hazard ratio [HR]: 0.32; 95% confidence interval [CI]: 0.08–0.88) than those on LMWH. Patients receiving DOACs for long-term therapy had a nonsignificantly lower rate of the composite outcome (HR: 0.70; 95% CI: 0.46–1.03) than those on VKAs. On multivariable analysis, patients initially receiving DOACs had a nonsignificantly lower risk for the composite outcome (HR: 0.36; 95% CI: 0.11–1.15) than those on LMWH, while those receiving DOACs for long-term therapy had a significantly lower risk (HR: 0.61; 95% CI: 0.41–0.92) than those on VKAs.

**Conclusions** Our data suggest that the use of DOACs may be more effective and safe than standard therapy in fragile patients with VTE, a subgroup of patients where the risk for bleeding is particularly high.

## Introduction

Subgroup analyses from randomized clinical trials suggested that the direct oral anticoagulants (DOACs) may have some advantages over standard therapy in fragile patients with venous thromboembolism (VTE). In the EINSTEIN trials, the rate of major bleeding was much lower in fragile patients receiving rivaroxaban than in those on standard therapy. 1 This difference was not seen in nonfragile patients. Besides, the HOKUSAI trial found a higher efficacy using edoxaban than warfarin in fragile patients, without any safety concern. 2 Fragile patients are underrepresented in clinical trials, and these favorable results have not been validated yet in real life. In a recent study, we found that 42% of VTE patients in real life are fragile, 3 and that during anticoagulation they had fewer VTE recurrences and more major bleeding events than the nonfragile.

The RIETE (Registro Informatizado de Enfermedad TromboEmbólica) registry is an ongoing, multicenter registry of consecutive patients with acute VTE with 223 collaborating centers in the Americas, Asia, and Europe (ClinicalTrials.gov identifier: NCT02832245). Since its inception in 2001, the aim of RIETE was to record data including the clinical characteristics, treatment patterns, and outcomes in patients diagnosed with VTE. 4 5 6 7 Using the RIETE database, the current study aimed to compare the outcomes during anticoagulation in fragile patients with VTE, according to the use of DOACs or standard anticoagulant therapy.

## Patients and Methods

Consecutive patients presenting with symptomatic, acute deep vein thrombosis (DVT) or pulmonary embolism (PE) confirmed by objective tests (compression ultrasonography or contrast venography for DVT; helical computed tomography [CT] scan, ventilation-perfusion lung scintigraphy, or angiography for PE) were enrolled in RIETE. Patients were excluded if they were currently participating in a therapeutic clinical trial with a blinded therapy. The rationale, design, and methodology of RIETE have been reported elsewhere. 8

### Study Design

We conducted a retrospective study of prospectively collected data from consecutive patients with acute VTE enrolled in the RIETE registry. Data were collected from January 2013 to April 2018, corresponding to the time when the prescription of DOACs was allowed. The major outcome was the composite of VTE recurrences or major bleeding events appearing during therapy. Comparisons were made separately for initial (DOACs vs. low-molecular-weight heparin [LMWH]) and for long-term therapy (DOACs vs. vitamin K antagonists [VKAs]).

For initial therapy, patients receiving DOACs were considered to receive the recommended therapy if they started within the first 48 hours after VTE diagnosis and were prescribed rivaroxaban 15 mg twice daily for 21 ± 2 days or apixaban 10 mg twice daily for 7 ± 2 days. For long-term therapy, patients were considered to receive the recommended doses if they were prescribed rivaroxaban 20 mg once daily; apixaban 5 mg twice daily; dabigatran 150 mg twice daily or edoxaban 60 mg once daily.

### Definitions

Fragile patients were defined as those having age ≥75 years, creatinine clearance (CrCl) levels ≤50 mL/min, or body weight ≤50 kg, as previously reported. Immobilized patients were defined as nonsurgical patients who had been immobilized (i.e., total bed rest with bathroom privileges) for ≥4 days in the 2-month period prior to VTE. Surgical patients were defined as those who underwent a surgical intervention in the 2 months prior to VTE. Active cancer was defined as newly diagnosed cancer, metastatic cancer, or cancer that was being treated (i.e., surgery, chemotherapy, radiotherapy, support therapy). Recent bleeding was defined as any major bleeding episode <30 days prior to VTE. Initial therapy was defined as any therapy administered during the first week in the case of LMWH or apixaban, and during the first 3 weeks in the case of rivaroxaban. Long-term therapy was defined as any therapy administered after the end of initial therapy. Bleeding events were classified as “major” if they were overt and required a transfusion of two units or more of blood, or were retroperitoneal, spinal or intracranial, or when they were fatal. Fatal bleeding was defined as any death occurring within 10 days of a major bleeding episode, in the absence of an alternative cause of death. Fatal PE, in the absence of autopsy, was defined as any death appearing within 10 days after symptomatic PE diagnosis, in the absence of any alternative cause of death.

### Treatment and Follow-up

Patients were managed according to each participating hospital's clinical practice, and there were no standardized or recommended duration of therapy or follow-up. All patients had to be followed up for at least 3 months in the outpatient clinic or physician's office. During each visit, any signs or symptoms suggesting VTE recurrences or bleeding complications were noted. Each episode of clinically suspected recurrent VTE was investigated by repeat compression ultrasonography, lung scanning, helical CT scan, or pulmonary angiography, as appropriate. Most outcomes were classified as reported by the clinical centers. However, if staff at the coordinating center were uncertain how to classify a reported outcome, that event was reviewed by a central adjudicating committee (less than 10% of events).

### Statistical Analysis

Categorical variables were compared using the chi-square test (two-sided) and Fisher's exact test (two-sided). Continuous variables were compared using Student *t* -test. Hazard ratios (HRs) and corresponding 95% confidence intervals (CIs) were calculated. Incidence rates were calculated as cumulative incidence (events/100 patient-years) and compared using the HRs and 95% CI. Cox proportional hazard models were used to compare the rates of the composite outcome of VTE recurrences or major bleeding occurring during initial and long-term therapy, separately. Crude and adjusted HRs as well as their 95% CIs were estimated. Covariates included in the adjusted model were those for which a statistically significant difference (a threshold *p* -value of 0.1 was set to assess significance of differences) was found between the different drugs, and a backward selection was used for the covariate selection in the multivariable model. Statistical analyses were conducted with SPSS for Windows Release 20.0 (SPSS Inc., Chicago, Illinois, United States).

### Role of the Funding Source

The sponsors of the RIETE registry (Sanofi and Bayer) had no role in study design, data collection, data analysis, data interpretation, or writing of the report. The corresponding author had full access to all the data in the study and had final responsibility for the decision to submit for publication.

## Results

**Fig. 1:**
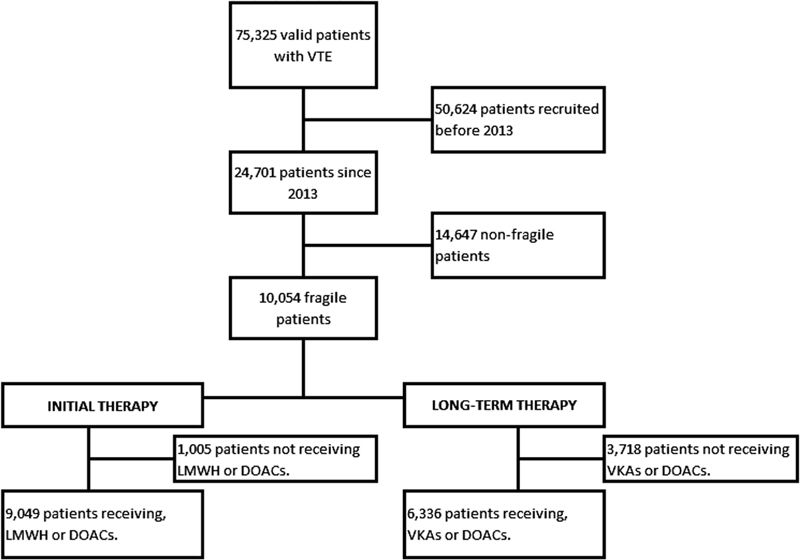
Flowchart of the patients.

From January 2013 to April 2018, 24,701 patients with acute VTE were recruited in RIETE. Of these, 10,054 (41%) were fragile ( Fig. 1 ). Initially, 473 fragile patients (4.7%) received DOACs (rivaroxaban 382, apixaban 91) and 8,577 (85%) were prescribed LMWH. Then, 1,298 patients (13%) switched to DOACs for long-term therapy (rivaroxaban 844, apixaban 344, dabigatran 69, edoxaban 41) and 5,038 (50%) to VKAs.

For initial therapy, patients receiving DOACs were 2 years younger than those treated with LMWH, less likely presented with PE (as compared with DVT), and were less likely to have cancer, anemia, or renal insufficiency, but were more likely to have unprovoked VTE, prior VTE, chronic heart failure, or to receive corticosteroids at baseline ( Table 1 ). Median duration of initial therapy with rivaroxaban, apixaban, or LMWH was 23, 9, and 10 days, respectively ( Table 2 ). Most patients received the recommended doses of DOACs (rivaroxaban 91%, apixaban 74%). Many patients receiving nonrecommended doses had recent major bleeding, severe renal insufficiency, liver failure, or thrombocytopenia. For long-term therapy, patients receiving DOACs were less likely to have renal insufficiency and more likely to have prior VTE, chronic heart failure, or recent major bleeding than those on long-term VKAs ( Table 1 ). Median duration of therapy with DOACs was half that of VKAs (96 vs. 172 days, respectively) and the proportion of patients receiving the recommended doses of DOACs was: rivaroxaban 65%, apixaban 68%, dabigatran 55%, and edoxaban 61%. Again, many patients receiving nonrecommended doses had recent major bleeding, severe renal insufficiency, liver failure, or thrombocytopenia.

**Table 1:** Clinical characteristics of the patients, according to initial therapy with LMWH or DOACs

	Initial therapy		Long-term therapy	
	DOACs	LMWH	DOACs	VKAs
Patients, *N*	473	8,577	1,298	5,038
Clinical characteristics				
Male gender	176 (37%)	3,291 (38%)	481 (37%)	1,917 (38%)
Mean age (years ± SD)	78 ± 11 a	80 ± 10	79 ± 9.9	80 ± 9.6
Age ≥ 75 y	417 (88%)	7,539 (88%)	1,170 (90%)	4,497 (89%)
Body weight (kg ± SD)	72 ± 14	71 ± 14	72 ± 15	72 ± 14
Body weight ≤ 50 kg	36 (7.6%)	780 (9.1%)	103 (7.9%)	324 (6.4%)
Initial VTE presentation				
Pulmonary embolism	242 (51%) a	4,932 (58%)	795 (61%)	3,156 (63%)
Risk factors for VTE				
Immobilization ≥ 4 d	133 (28%)	2,229 (26%)	307 (24%)	1,162 (23%)
Surgery	40 (8.5%)	661 (7.7%)	103 (7.9%)	361 (7.2%)
Cancer	66 (14%) b	2,168 (25%)	157 (12%)	626 (12%)
Estrogen use	12 (2.5%)	292 (3.4%)	41 (3.2%)	129 (2.6%)
Pregnancy/postpartum	0	14 (0.16%)	1 (0.08%)	6 (0.12%)
None of the above	251 (53%) c	4,060 (47%)	755 (58%)	3,011 (60%)
Prior VTE	83 (18%) c	1,143 (13%)	232 (18%) a	739 (15%)
Underlying diseases				
Chronic heart failure	79 (17%) b	932 (11%)	184 (14%) b	543 (11%)
Chronic lung disease	77 (16%)	1,288 (15%)	183 (14%)	786 (16%)
Recent major bleeding	9 (1.9%)	215 (2.5%)	38 (2.9%) b	56 (1.1%)
Blood tests at baseline				
Anemia	144 (31%) b	3,488 (41%)	420 (32%)	1,649 (33%)
Platelet count < 100,000/µL	5 (1.1%) c	238 (2.8%)	17 (1.3%)	109 (2.2%)
CrCl levels (mL/min ± SD)	60 ± 21 b	55 ± 24	59 ± 21 b	54 ± 23
CrCl levels ≤50 mL/min	169 (36%) b	4,185 (49%)	496 (38%) b	2,479 (49%)
Concomitant drugs				
Corticosteroids	74 (16%) a	977 (11%)	135 (10%)	512 (10%)
Antiplatelets	134 (28%)	2,300 (27%)	340 (26%)	1,362 (27%)
NSAIDs	38 (8.1%)	665 (7.8%)	98 (7.6%)	390 (7.7%)

Abbreviations: CrCl, creatinine clearance; DOACs, direct oral anticoagulants; LMWH, low-molecular-weight heparin; NSAIDs, nonsteroidal anti-inflammatory drugs; SD, standard deviation; VKAs, vitamin K antagonists; VTE, venous thromboembolism.

Comparisons between DOACs versus standard therapy:

a *p* < 0.01;

b *p* < 0.001;

c *p* < 0.05.

**Table 2:** Treatment strategies for initial and for long-term therapy

	Initial therapy		Long-term therapy	
	DOACs	LMWH	DOACs	VKAs
Initial therapy with LMWH, *N*	473	8,577	1,298	5,038
Mean days of therapy ( ± SD)	20 ± 16 a	13 ± 16	149 ± 156	251 ± 248
Median days of therapy (IQR)	23 (12–24) a	10 (7–13)	96 (65–183)	172 (99–313)
Mean LMWH doses (IU/kg/d)	–	170 ± 46	–	–
Rivaroxaban, *N*	382		844	
Mean days of therapy ( ± SD)	21 ± 17	–	144 ± 163	–
Median days of therapy (IQR)	23 (14–24)	–	94 (42–172)	–
30 mg daily	346 (91%)	–	204 (24%)	–
20 mg daily	10 (2.6%)	–	547 (65%)	–
15 mg daily	25 (6.5%)	–	87 (10%)	–
Apixaban, *N*	91		344	
Mean days of therapy ( ± SD)	10 ± 11	–	162 ± 147	–
Median days of therapy (IQR)	9 (9–10)	–	114 (85–191)	–
20 mg daily	67 (74%)	–	44 (13%)	–
10 mg daily	13 (14%)	–	233 (68%)	–
5 mg daily	11 (12%)	–	64 (19%)	–
Dabigatran, *N*			69	
Mean days of therapy ( ± SD)	–	–	173 ± 137	–
Median days of therapy (IQR)	–	–	134 (93–203)	–
220 mg daily	–	–	38 (55%)	–
Other doses/not reported	–	–	31 (45%)	–
Edoxaban, *N*			41	
Mean days of therapy ( ± SD)	–	–	125 ± 78	–
Median days of therapy (IQR)	–	–	98 (88–170)	–
60 mg daily	–	–	25 (61%)	–
30 mg daily	–	–	16 (39%)	–

Abbreviations: DOACs, direct oral anticoagulants; IQR, interquartile range; LMWH, low-molecular-weight heparin; SD, standard deviation; VKAs, vitamin K antagonists.

Comparisons between DOACs versus standard therapy: ^a^ *p* < 0.001.

**Fig. 2:**
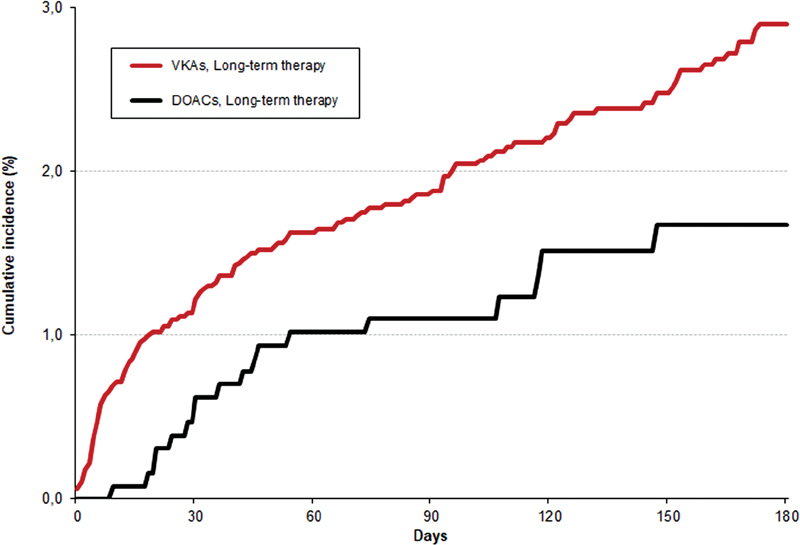
Cumulative rates of the composite outcome during the first 6 months of therapy.

During the course of anticoagulation, 95 patients developed VTE recurrences (LMWH 85, DOACs 10), 262 had major bleeding (VKAs 241, DOACs 21), and 595 died (standard therapy 536, DOACs 59). Patients receiving DOACs for initial therapy had a significantly lower rate of the composite outcome (HR: 0.32; 95% CI: 0.08–0.88) and a lower mortality rate (HR: 0.29; 95% CI: 0.12–0.62) than those receiving LMWH ( Table 3 ). No patient receiving DOACs initially developed VTE recurrences, and the rate of major bleeding was nonsignificantly lower (HR: 0.37; 95% CI: 0.09–1.04) than in those on LMWH. All three major bleeding events occurring in patients initially receiving DOACs appeared in patients aged ≥75 years, with CrCl levels >50 mL/min. None of the 169 patients with CrCl levels ≤50 mL/min developed major bleeding during initial therapy with DOACs. No patients initially receiving DOACs died of PE or bleeding. On multivariable analysis, patients initially receiving DOACs had a nonsignificantly lower risk for the composite outcome (HR: 0.36; 95% CI: 0.11–1.15) than those on LMWH. During long-term therapy, patients receiving DOACs had a nonsignificantly lower rate of the composite outcome (HR: 0.70; 95% CI: 0.46–1.03), VTE recurrences (HR: 0.77; 95% CI: 0.38–1.45), or major bleeding (HR: 0.68; 95% CI: 0.41–1.10) than those on VKAs, and a similar mortality rate (HR: 1.03; 95% CI: 0.76–1.39) ( Fig. 2 and Tables 3 and 4 ). Most patients (17 of 18, 94%) presenting with major bleeding during long-term therapy with DOACs were aged ≥75 years. Of these, eight patients had CrCl levels >50 mL/min and nine had levels ≤50 mL/min. On multivariable analysis, patients receiving DOACs had a significantly lower risk for the composite outcome (HR: 0.61; 95% CI: 0.41–0.92) than those on VKAs ( Table 5 ).

**Table 3:** Outcomes in fragile patients with VTE, according to initial therapy with LMWH or DOACs

	*N*	*N* per 100 patient-years	*N*	*N* per 100 patient-years	Hazard ratio (95% CI)
Initial therapy	DOACs		LMWH		
Patients, *N*	473		8,577		
Recurrent DVT	0	–	8	2.29 (1.07–4.36)	–
Recurrent PE	0	–	12	3.44 (1.86–5.84)	–
Recurrent VTE	0	–	20	5.74 (3.60–8.71)	–
Major bleeding	3	11.7 (2.98–31.9)	109	31.4 (25.9–37.7)	0.37 (0.09–1.04)
Gastrointestinal	1	3.90 (0.20–19.2)	30	8.60 (5.91–12.1)	0.45 (0.02–2.38)
Intracranial	1	3.90 (0.19–19.2)	9	2.58 (1.26–4.73)	1.52 (0.07–9.09)
Composite outcome	3	11.7 (2.98–31.9)	128	36.9 (30.9–43.8)	0.32 (0.08–0.88)
Death	6	23.4 (9.49–48.7)	277	79.7 (70.8–89.6)	0.29 (0.12–0.62)
Causes of death					
Pulmonary embolism	0	–	54	15.5 (11.7–20.0)	–
Bleeding	0	–	16	4.58 (2.71–7.28)	–
**Long-term therapy**	**DOACs**		**VKAs**		
Patients, *N*	1,298		5,038		
Recurrent DVT	3	0.40 (0.10–1.09)	31	0.82 (0.57–1.16)	0.49 (0.12–1.43)
Recurrent PE	7	0.94 (0.41–1.85)	35	0.93 (0.66–1.28)	1.01 (0.41–2.17)
Recurrent VTE	10	1.34 (0.68–2.39)	65	1.74 (1.35–2.20)	0.77 (0.38–1.45)
Major bleeding	18	2.41 (1.47–3.74)	132	3.52 (2.95–4.16)	0.68 (0.41–1.10)
Gastrointestinal	12	1.61 (0.87–2.73)	54	1.43 (1.09–1.86)	1.12 (0.57–2.04)
Intracranial	2	0.27 (0.04–0.88)	20	0.53 (0.33–0.80)	0.51 (0.08–1.85)
Composite outcome	27	3.63 (2.44–5.21)	193	5.19 (4.50–5.96)	0.70 (0.46–1.03)
Death	53	7.09 (5.36–9.20)	259	6.86 (6.06–7.73)	1.03 (0.76–1.39)
Causes of death					
Recurrent PE	3	0.40 (0.10–1.09)	2	0.05 (0.01–0.17)	7.69 (1.12–50.0)
Bleeding	1	0.13 (0.01–0.66)	19	0.50 (0.31–0.77)	0.27 (0.01–1.45)
Fatal PE or bleeding	4	0.53 (0.17–1.29)	21	0.56 (0.35–0.84)	0.96 (0.28–2.63)

**Abbreviations:** DOACs, direct oral anticoagulants; DVT, deep vein thrombosis; LMWH, low-molecular-weight heparin; PE, pulmonary embolism; VKAs, vitamin K antagonists; VTE, venous thromboembolism.

**Table 4:** Cumulative rates of the composite of VTE recurrences or major bleeding during the first 6 months of therapy

Days		30	60	90	120	150	180
VKAs	Patients at risk	4,988	4,827	4,616	3,935	3,176	2,767
	Events	61 (1.22%)	81 (1.63%)	93 (1.88%)	106 (2.21%)	115 (2.48%)	127 (2.9%)
DOACs	Patients at risk	1,288	1,250	1,118	857	653	544
	Events	8 (0.62%)	13 (1.02%)	14 (1.11%)	17 (1.51%)	18 (1.67%)	18 (1.67%)

**Table 5:** Uni- and multivariable analyses for the composite outcome of VTE recurrences plus major bleeding

	Initial therapy		Long-term therapy	
	Univariable	Multivariable	Univariable	Multivariable
Events, *N*	131			
Clinical characteristics				
Male gender	0.73 (0.51–1.06)	0.70 (0.48–1.02)	1.03 (0.78–1.35)	–
Age > 80 y	1.16 (0.82–1.64)	–	1.43 (1.09–1.87) a	1.31 (0.99–1.73)
Body weight ≤ 70 kg	0.90 (0.64–1.27)	–	1.32 (1.01–1.72) b	1.25 (0.95–1.65)
Initial VTE presentation				
Pulmonary embolism	1.34 (0.94–1.92)	–	1.13 (0.85–1.50)	–
Risk factors for VTE				
Unprovoked	Ref. c	Ref. b	Ref. c	Ref. b
Cancer	1.90 (1.23–2.95) a	1.71 (1.09–2.69) b	1.67 (1.15–2.44) a	1.52 (1.04–2.23) b
Transient risk factors	2.17 (1.43–3.28) c	1.80 (1.18–2.76) a	1.65 (1.22–2.21) c	1.36 (1.01–1.85) b
Prior VTE	1.11 (0.69–1.79)	–	1.12 (0.80–1.58)	–
Underlying diseases				
Chronic heart failure	1.21 (0.73–2.01)	–	1.56 (1.08–2.23) b	1.37 (0.94–1.98)
Chronic lung disease	1.33 (0.86–2.06)	–	1.26 (0.89–1.78)	–
Recent major bleeding	4.51 (2.59–7.86) c	3.69 (2.09–6.54) c	3.28 (1.62–6.65) a	2.37 (1.15–4.88) b
Blood tests				
Anemia	1.52 (1.08–2.14) b	1.23 (0.86–1.76)	1.96 (1.50–2.55) c	1.69 (1.28–2.23) c
Platelet count < 100,000/µL	1.52 (0.62–3.72)	–	1.52 (0.72–3.23)	–
CrCl levels ≤50 mL/min	1.12 (0.79–1.58)	–	1.26 (0.97–1.64)	1.02 (0.77–1.35)
Concomitant drugs				
Corticosteroids	1.28 (0.79–2.08)	–	1.47 (1.00–2.18)	1.26 (0.85–1.87)
Antiplatelets	1.45 (1.01–2.09) b	1.48 (1.03–2.13) b	1.32 (0.99–1.75)	1.18 (0.88–1.57)
NSAIDs	1.30 (0.73–2.30)	–	1.78 (1.21–2.63) a	1.70 (1.15–2.53) a
DOACs				
Yes	0.34 (0.11–1.06)	0.36 (0.11–1.15)	0.65 (0.43–0.97) b	0.61 (0.41–0.92) b

Abbreviations: CrCl, creatinine clearance; DOACs, direct oral anticoagulants; NSAIDs, nonsteroidal anti-inflammatory drugs; Ref., reference; VTE, venous thromboembolism.

Comparisons: ^a^ *p* < 0.01; ^b^ *p* < 0.05; ^c^ *p* < 0.001.

During initial therapy, the rate of major bleeding in patients receiving DOACs at recommended doses was lower than in those receiving LMWH (HR: 0.14; 95% CI: 0.007–0.71), as shown in Table 6 . The rate in patients receiving nonrecommended doses of DOACs was higher. During long-term therapy, patients receiving DOACs at recommended doses (but not those on nonrecommended doses) had half the rate of the composite outcome (HR: 0.45; 95% CI: 0.23–0.80) compared to those on VKAs. The rates of the composite outcome during long-term therapy with rivaroxaban, apixaban, or dabigatran were similar.

**Table 6:** Rates of VTE recurrences, major bleeding, and the composite outcome, according to prescribed drugs and doses. Results expressed as number of events per 100 patient-years and 95% confidence intervals

	Initial therapy		Long-term therapy	
	*N*	Events per 100 patient-years	*N*	Events per 100 patient-years
Standard therapy, *N*	8,577		5,038	
VTE recurrences	20	5.74 (3.60–8.71)	65	1.74 (1.35–2.20)
Major bleeding	109	31.4 (25.9–37.7)	132	3.52 (2.85–4.16)
Composite outcome	128	36.9 (30.9–43.8)	193	5.19 (4.50–5.96)
DOACs, recommended doses, *N*	413		843	
VTE recurrences	0	–	4	0.85 (0.27–2.06)
Major bleeding	1	4.44 (0.22–21.9)	7	1.49 (0.65–2.95)
Composite outcome	1	4.44 (0.22–21.9)	11	2.35 (1.23–4.08)
DOACs, nonrecommended doses, *N*	60		455	
VTE recurrences	0	–	6	2.19 (0.89–4.56)
Major bleeding	2	65.4 (11.0–216.1)	11	4.02 (2.12–6.99)
Composite outcome	2	65.4 (11.0–216.1)	16	5.88 (3.48–9.35)
Rivaroxaban, *N*	382		844	
VTE recurrences	0	–	9	1.85 (0.90–3.39)
Major bleeding	3	13.2 (3.37–36.1)	10	2.05 (1.04–3.65)
Composite outcome	3	13.2 (3.37–36.1)	18	3.71 (2.27–5.74)
Apixaban, *N*	91		344	
VTE recurrences	0	–	1	0.51 (0.03–2.50)
Major bleeding	0	–	6	3.05 (1.24–6.34)
Composite outcome	0	–	7	3.56 (1.56–7.04)
Dabigatran, *N*	0		69	
VTE recurrences	0	–	0	–
Major bleeding	0	–	2	4.84 (0.81–16.0)
Composite outcome	0	–	2	4.84 (0.81–16.0)
Edoxaban, *N*	0		41	
VTE recurrences	0	–	0	–
Major bleeding	0	–	0	–
Composite outcome	0	–	0	

Abbreviations: DOACs, direct oral anticoagulants; VTE, venous thromboembolism.

## Discussion

Our data, obtained from a series of fragile patients with VTE in real life, suggest that the use of DOACs may be more effective and safe than standard therapy in this patient population. No patient receiving DOACs for initial therapy in our cohort developed VTE recurrences, and their rate of major bleeding was one-third the rate in patients on LMWH. On multivariable analysis, the risk for the composite outcome was not significantly lower, most likely since fragile patients on DOACs were younger and less likely to have cancer, anemia, or renal insufficiency than those on LMWH. During long-term therapy however, the clinical characteristics of patients receiving DOACs or VKAs were similar, and the use of DOACs was associated with a lower risk for the composite outcome after adjusting for potential confounders.

In a previous study using the RIETE database, we found that fragile patients with VTE had a twofold higher rate of major bleeding and half the rate of VTE recurrences compared to the nonfragile. 3 Thus, there are reasons to suggest that when choosing an anticoagulant drug for fragile patients with VTE, safety is an important issue. The current findings confirm that the rate of major bleeding during the course of anticoagulation clearly outweighed the rate of VTE recurrences (262 vs. 95 events, respectively). Unexpectedly however, only one in every 20 fragile patients was prescribed DOACS initially, and only one in every eight for long-term therapy. Data from the PREFER in VTE registry also showed that the use of DOACs was less likely in elderly patients or in those with renal insufficiency. 1 9 10 11 12 13 14 It would seem to be more cost effective to prescribe safer drugs in patients at increased risk for bleeding, as fragile patients are. As far as we know, no studies have compared yet the efficacy and safety of the DOACs versus standard therapy in fragile patients with VTE. In a recent study, Coleman et al compared rivaroxaban versus VKAs in a cohort of frail patients with VTE. 15 However, the term “fragile” has been recently incorporated into the literature to include VTE patients who are elderly, renally impaired, or with low body weight. 1 This term should not be confused with “frail,” which usually refers to elderly people with reduced physiologic reserve associated with increased susceptibility to disability. 16 17 18 19 20

During long-term therapy, only two in every three patients (65%) on DOACs received the recommended doses. This is important since the use of DOACs at nonrecommended doses has been associated with a much higher rate of VTE recurrences and a similar rate of major bleeding compared to those on recommended doses. 21 One in every six patients (496 of 2,975, 17%) with CrCl levels <50 mL/min in our series was prescribed DOACs, a subgroup of patients at increased risk for bleeding. 4 22 23 However, only six of these patients had major bleeding, thus suggesting that the DOACs might be at least as safe as VKAs in patients with CrCl levels <50 mL/min. Unfortunately, randomized clinical trials to compare the DOACs versus standard therapy are not allowed in patients with CrCl levels <50 mL/min.

The present study has several potential limitations. First, since RIETE is an observational registry (and not a randomized trial) our data are hypothesis-generating. They might be a useful basis for future controlled clinical trials comparing different therapeutic strategies, but we should be extremely cautious in suggesting changes in treatment strategies just because of uncontrolled registry data. Second, for long-term therapy we compared DOACs versus VKAs, but one in every eight such patients (12%) had active cancer, and they should have been compared rather with LMWH. Third, treatment varied with local practice, and is likely to have been influenced by a physician's assessment of a patient's risk of bleeding. Fourth, patients in the RIETE database were selected from several different countries. The variability of practices in different countries could potentially affect the study outcomes. Fifth, a variety of practitioners entered data into the registry, which may lend itself to potential inaccuracies in the data being reported. Finally, lack of central adjudication of the events is another limitation that is impossible for us to overcome. The main strengths of our observation are the high number of included patients, the strict diagnostic criteria, and the reporting of objectively established outcomes (major bleeding and recurrent VTE). Additionally, the population-based sample we used describes the effects of anticoagulant therapy in “real-world” clinical care, as opposed to that in a protocol driven randomized trial, and enhances the generalizability of our findings.

In summary, in real life 41% of VTE patients were fragile, a subgroup of patients where the risk for bleeding is particularly high. Our findings suggest that the use of DOACs may be more effective and safe than standard therapy in this patient population. Intervention studies specifically designed to confirm our findings and the potential role of the DOACs in fragile patients receiving anticoagulant therapy for VTE are warranted.

## Notes

Appendix A
**Coordinator of the RIETE Registry:** Manuel Monreal.

**RIETE Steering Committee Members:** Paolo Prandoni, Benjamin Brenner, and Dominique Farge-Bancel.

**RIETE National Coordinators:** Raquel Barba (Spain), Pierpaolo Di Micco (Italy), Laurent Bertoletti (France), Sebastian Schellong (Germany), Inna Tzoran (Israel), Abilio Reis (Portugal), Marijan Bosevski (R. Macedonia), Henri Bounameaux (Switzerland), Radovan Malý (Czech Republic), Peter Verhamme (Belgium), Joseph A. Caprini (United States), Hanh My Bui (Vietnam).

**RIETE Registry Coordinating Center:** S&H Medical Science Service.

**Members of the RIETE Group:** SPAIN: Adarraga MD, Agud M, Aibar MA, Aibar J, Alcalde-Manero M, Amado C, Arcelus JI, Ballaz A, Barba R, Barrón M, Barrón-Andrés B, Bascuñana J, Blanco-Molina A, Camon AM, Cañas I, Carrasco C, Castro J, de Ancos C, del Toro J, Demelo P, Díaz-Pedroche C, Díaz-Peromingo JA, Falgá C, Farfán AI, Fernández-Capitán C, Fernández-Criado MC, Fidalgo MA, Font C, Font L, Furest I, García MA, García-Bragado F, García-Morillo M, García-Raso A, García-Ródenas M, Gavín O, Gaya-Manso I, Gayol MC, Gil-Díaz A, Gómez V, Gómez-Cuervo C, González-Martínez J, Grau E, Gutiérrez J, Hernández-Blasco LM, Iglesias M, Jara-Palomares L, Jaras MJ, Jiménez D, Jou I, Joya MD, Lima J, Llamas P, Lobo JL, López-Jiménez L, López-Miguel P, López-Núñez JJ, López-Reyes R, López-Sáez JB, Lorente MA, Lorenzo A, Loring M, Madridano O, Maestre A, Marchena PJ, Martín del Pozo M, Martín-Fortea MP, Martín-Guerra JM, Martínez-González L, Mella C, Mellado M, Monreal M, Montesa C, Morales MV, Nieto MA, Nieto JA, Núñez MJ, Olivares MC, Otero R, Pedrajas JM, Pellejero G, Pérez-Ductor C, Pérez-Jacoiste A, Pérez-Rus G, Peris ML, Porras JA, Rivas A, Rodríguez-Hernández A, Rubio CM, Ruiz-Artacho P, Ruiz-Ruiz J, Ruiz-Sada P, Sahuquillo JC, Sala-Sainz MC, Salazar V, Salgueiro G, Sampériz A, Sánchez-Muñoz-Torrero JF, Sancho T, Soler S, Suriñach JM, Tolosa C, Torres MI, Trujillo-Santos J, Uresandi F, Valle R, Vidal G, Villares P. ARGENTINA: Gutiérrez P, Vázquez FJ, Vilaseca A. BELGIUM: Vanassche T, Vandenbriele C, Verhamme P. CZECH REPUBLIC: Hirmerova J, Malý R. ECUADOR: Salgado E. FRANCE: Benzidia I, Bertoletti L, Bura-Riviere A, Debourdeau P, Farge-Bancel D, Hij A, Mahé I, Merah A, Moustafa F. GERMANY: Schellong S. ISRAEL: Braester A, Brenner B, Ellis M, Tzoran I. IRAN: Sharif-Kashani B. ITALY: Barillari G, Bilora F, Bortoluzzi C, Brandolin B, Ceccanti G, Ciammaichella M, Dentali F, Di Micco P, Grandone E, Maida R, Mastroiacovo D, Pallotti G, Pace F, Parisi R, Pesavento R, Prandoni P, Quintavalla R, Rocci A, Siniscalchi C, Tufano A, Visonà A, Vo Hong N, Zalunardo B. LATVIA: Gibietis V, Kigitovica D, Skride A. REPUBLIC OF MACEDONIA: Bosevski M. SWITZERLAND: Bounameaux H, Mazzolai L. USA: Caprini J. VIETNAM: Bui HM, Pham KQ.

## References

[1] Bauersachs R Berkowitz S D Brenner B Oral rivaroxaban for symptomatic venous thromboembolism N Engl J Med 2010 363 26 2499 2510 21128814 10.1056/NEJMoa1007903

[2] Büller H R Décousus H Grosso M A Edoxaban versus warfarin for the treatment of symptomatic venous thromboembolism N Engl J Med 2013 369 15 1406 1415 23991658 10.1056/NEJMoa1306638

[3] Moustafa F Pierfranceschi M G Di Micco P Clinical outcomes during anticoagulant therapy in fragile patients with venous thromboembolism Res Pract Thromb Haemost 2017 1 02 172 179 30046687 10.1002/rth2.12036 PMC6058265

[4] Ruíz-Giménez N Suárez C González R Predictive variables for major bleeding events in patients presenting with documented acute venous thromboembolism. Findings from the RIETE Registry Thromb Haemost 2008 100 01 26 31 18612534 10.1160/TH08-03-0193

[5] Muñoz-Torrero J F Bounameaux H Pedrajas J M Effects of age on the risk of dying from pulmonary embolism or bleeding during treatment of deep vein thrombosis J Vasc Surg 2011 54 06, Suppl):26S 32S 10.1016/j.jvs.2011.05.114 21908150

[6] Jiménez D de Miguel-Díez J Guijarro R Trends in the management and outcomes of acute pulmonary embolism: Analysis from the RIETE Registry J Am Coll Cardiol 2016 67 02 162 170 26791063 10.1016/j.jacc.2015.10.060

[7] Muriel A Jiménez D Aujesky D Survival effects of inferior vena cava filter in patients with acute symptomatic venous thromboembolism and a significant bleeding risk J Am Coll Cardiol 2014 63 16 1675 1683 24576432 10.1016/j.jacc.2014.01.058

[8] Bikdeli B Jimenez D Hawkins M Rationale, design and methodology of the computerized registry of patients with venous thromboembolism (RIETE) Thromb Haemost 2018 118 01 214 224 29304541 10.1160/TH17-07-0511 PMC5821113

[9] Bauersachs R Agnelli G Gitt A K The role of heparin lead-in in the real-world management of acute venous thromboembolism: the PREFER in VTE registry Thromb Res 2017 157 181 188 28780343 10.1016/j.thromres.2017.07.029

[10] Cohen A T Gitt A K Bauersachs R The management of acute venous thromboembolism in clinical practice. Results from the European PREFER in VTE Registry Thromb Haemost 2017 117 07 1326 1337 10.1160/TH16-10-0793 PMC6291854 28405675

[11] Schulman S Kearon C Kakkar A K Dabigatran versus warfarin in the treatment of acute venous thromboembolism N Engl J Med 2009 361 24 2342 2352 19966341 10.1056/NEJMoa0906598

[12] Büller H R Prins M H Lensin A W Oral rivaroxaban for the treatment of symptomatic pulmonary embolism N Engl J Med 2012 366 14 1287 1297 22449293 10.1056/NEJMoa1113572

[13] Agnelli G Buller H R Cohen A Oral apixaban for the treatment of acute venous thromboembolism N Engl J Med 2013 369 09 799 808 23808982 10.1056/NEJMoa1302507

[14] Becattini C Agnelli G Treatment of venous thromboembolism with new anticoagulant agents J Am Coll Cardiol 2016 67 16 1941 1955 27102510 10.1016/j.jacc.2016.01.072

[15] Coleman C I Turpie A GG Bunz T J Beyer-Westendorf J Effectiveness and safety of rivaroxaban versus warfarin in frail patients with venous thromboembolism Am J Med 2018 131 08 9330 9380 10.1016/j.amjmed.2018.02.015 29526541

[16] Buchner D M Wagner E H Preventing frail health Clin Geriatr Med 1992 8 01 1 17 1576567

[17] Rockwood K Song X MacKnight C A global clinical measure of fitness and frailty in elderly people CMAJ 2005 173 05 489 495 16129869 10.1503/cmaj.050051 PMC1188185

[18] Robinson T N Eiseman B Wallace J I Redefining geriatric preoperative assessment using frailty, disability and co-morbidity Ann Surg 2009 250 03 449 455 19730176 10.1097/SLA.0b013e3181b45598

[19] Robinson T N Wu D S Pointer L Dunn C L Cleveland J C Jr Moss M Simple frailty score predicts postoperative complications across surgical specialties Am J Surg 2013 206 04 544 550 23880071 10.1016/j.amjsurg.2013.03.012 PMC3788864

[20] Perera V Bajorek B V Matthews S Hilmer S N The impact of frailty on the utilisation of antithrombotic therapy in older patients with atrial fibrillation Age Ageing 2009 38 02 156 162 19151165 10.1093/ageing/afn293

[21] Trujillo-Santos J Di Micco P Dentali F Real-life treatment of venous thromboembolism with direct oral anticoagulants: the influence of recommended dosing and regimens Thromb Haemost 2017 117 02 382 389 27786333 10.1160/TH16-07-0494

[22] Monreal M Falgá C Valle R Venous thromboembolism in patients with renal insufficiency: findings from the RIETE Registry Am J Med 2006 119 12 1073 1079 17145252 10.1016/j.amjmed.2006.04.028

[23] Moustafa F Stehouwer A Kamphuisen P Management and outcome of major bleeding in patients receiving vitamin K antagonists for venous thromboembolism Thromb Res 2018 171 74 80 30265883 10.1016/j.thromres.2018.09.049

